# Association of chronic kidney disease with periprocedural myocardial injury after elective stent implantation: A single center prospective cohort study

**DOI:** 10.1097/MD.0000000000005381

**Published:** 2016-11-11

**Authors:** Helena Jerkic, Tomislav Letilovic, Mario Stipinovic, Darko Pocanic, Jasmina Catic, Mladen Knotek

**Affiliations:** a Division of Cardiology; b Division of Nephrology, Department of Medicine, University Hospital Merkur; c Division of Cardiology, Department of Medicine, University Hospital Dubrava; d University of Zagreb Medical School, Zagreb, Croatia.

**Keywords:** chronic kidney disease, coronary artery disease, percutaneous coronary intervention, periprocedural myocardial injury

## Abstract

Coronary artery disease (CAD) is the leading cause of mortality in patients with chronic kidney disease (CKD). Patients with CKD who undergo percutaneous coronary intervention (PCI) may have more ischemic events than patients without CKD. The aim of our study was to determine the incidence of periprocedural myocardial injury (PMI) after elective stent implantation in patients with CKD using the Third Joint ESC/ACCF/AHA/WHF PMI definition.

In a single center prospective cohort study, we enrolled 344 consecutive patients who underwent elective PCI in a period of 39 months. Serum troponin I (cTnI) concentrations were measured at baseline and at 8 and 16 hours after PCI. Periprocedural increase of cTnI, according to the most recent PMI definition, was used to define both the presence and intensity of PMI. Patients were further stratified according to the estimated glomerular filtration rate (eGFR) using 4 variable Modification of Diet in Renal Disease (MDRD) equation: control group with eGFR >90 mL/min/1.73 m^2^ and the CKD group with eGFR < 90 mL/min/1.73 m^2^, with further subdivision according to the CKD stage.

We found no significant difference in the incidence as well as intensity of the PMI in the control (>90 mL/min/1.73 m^2^) and the CKD group (<90 mL/min/1.73 m^2^) both 8 and 16 hours after PCI. When the CKD patients were further subdivided according to their CKD stage, there was again no difference in the intensity or incidence of PMI compared to the control group. Further analyses of our data showed angina pectoris CCS IV, bare metal stent (BMS) implantation, and treatment with angiotensin-converting enzyme inhibitors (ACEI) as independent predictors of PMI. Furthermore, the presence of hypertension was inversely related to the occurrence of PMI.

Applying the new guidelines for PMI and using the eGFR equation most suitable for our patients, we found no association between PMI and CKD. Further analyses showed other factors that could potentially influence the occurrence of PMI.

## 1 Introduction

Coronary artery disease (CAD) is the leading cause of death in patients with chronic kidney disease (CKD).^[1]^ Dialysis patients experience a mortality rate after myocardial infarction that is up to 15 times higher than in patients with normal renal function. Mortality at 1 year after myocardial infarction can reach as high as 59% in the dialysis population.^[2]^ The excess cardiovascular risk with CKD is explained by higher prevalence of well-established cardiovascular risk factors such as hypertension, diabetes mellitus, dyslipidaemia, and uremia, which all promote rapid progression of CAD.^[3]^

Patients with CKD have been reported to have elevated risk of adverse clinical outcomes after coronary revascularization, including higher incidence of myocardial infarction, as compared to non-CKD patients.^[4]^ In a largest study to date examining risk factors for in-hospital mortality following PCI in 25018 patients, CKD, especially end-stage renal disease (ESRD), was found to be independently associated with increased in-hospital all-cause mortality. Independent risk factors in patients with ESRD included myocardial infarction within 72 hours, while emergency PCI was associated with increased risk of death in patients with moderate CKD.^[5]^

Although the PCI may be a high-risk procedure in CKD, PCI was associated with lower risk of death in end-stage renal disease patients, compared with no revascularization.^[6]^ Thus, the presence of kidney disease or dependence on dialysis should not be the barrier to PCI revascularization in patients with acute coronary syndrome.

However, despite prevalence of reports on adverse association of CKD with PCI outcome in the acute setting (i.e. for acute coronary syndrome), there is still a shortage of studies addressing effect of CKD burden on early outcomes of elective PCI. Therefore, our study was designed to test the hypothesis that presence of CKD and its burden (CKD grade) would be associated with increased risk of PMI in elective PCI patients.

## 2 Methods

This study prospectively included 344 consecutive patients with stable angina pectoris who underwent an elective PCI at Merkur University Hospital, Zagreb, Croatia. Enrollment period was 39 months, that is, between March 2012 and June 2015. This study was approved by the Merkur Hospital ethics committee and all patients provided informed consent. The primary endpoint was incidence of PMI, depending on eGFR. All patients had stable angina pectoris with documented inducible myocardial ischemia. Stable patients were defined as those with no recent deterioration of pain in the previous 2 months or without rest angina in the previous 48 hours. Further criteria for inclusion were that the PCI procedure was successful and an optimal final result was obtained, that is, a thrombolysis in myocardial infarction (TIMI) flow grade 3 in the treated vessel with a residual stenosis <20% by quantitative coronary angiography (QCA).

The exclusion criteria were: age <18, acute coronary syndrome, acute kidney injury, multivessel stenting in a single procedure, major (>1.5 mm) side branch occlusion, chronic total occlusion, major hemorrhage within 4 weeks or contraindication to the use dual antiplatelet therapy, unsuccessful procedures, and target lesion in saphenous graft. All patients received a dual antiplatelet therapy with aspirin 100 mg/day and clopidogrel 75 mg/day for 5 days before elective PCI. Immediately before PCI procedures, a bolus of unfractionated heparin according to a standardized protocol was administered. PCI procedures were performed using either of 2 types of low osmolar contrast media, iodixanol, or ioversol.

Serum TnI levels were measured using a chemiluminescent microparticle immunoassay technology (CMIA) on the ARCHITECT I System with STAT protocol capability (Abbott Laboratories, Abbott Park, IL 60064). All blood samples have been tested with the same reagents kit (ARCHITECT STAT Troponin-I Reagent Kit).

Blood samples were collected immediately before PCI and at 8 and 16 hours after PCI.

The upper reference limit (URL) of serum cTnI was 0.04 ng/mL. PMI was defined as an increase in cTnI at 8 or 16 hours after PCI to a concentration of >0.04 ng/mL. We further divided PMI into 2 groups. If cTnI increase <5× URL, it was considered a PMI of low degree, whereas an increase to ≥ 5× was considered a PMI of high degree (Table 1). If basal cTnI was >URL, then an increase of >20% of basal value was considered a PMI of high degree, and an increase of <20% of basal value was considered a PMI of low degree (Table 1).

**Table 1:**
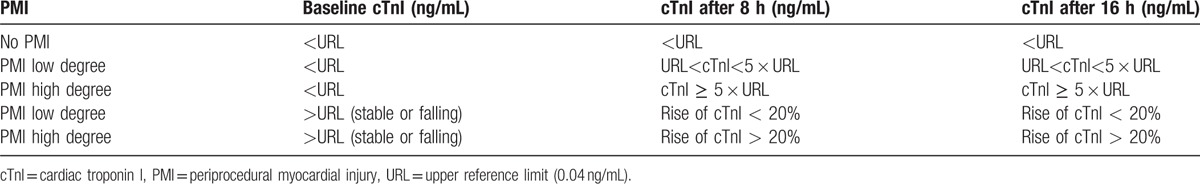
Definition of high and low degree periprocedural myocardial injury.

This classification and cut-off values were derived from the most recent ESC Consensus document on myocardial infarction.^[7]^

Patients were divided into 2 groups according to the eGFR. The control group comprised of patients with eGFR ≥90 mL/min/1.73 m^2^ and in the CKD group there were patients with eGFR <90 mL/min/1.73 m^2^. The CKD group was further subdivided into 4 groups depending on the CKD stage: (1) eGFR 60 to 89 mL/min/1.73 m^2^, (2) eGFR 30 to 59 mL/min/1.73 m^2^, (3) eGFR 15 to 29 mL/min/1.73 m^2^, (4) eGFR <15 mL/min/1.73 m^2^. We have used CKD classification based on 5 categories of eGFR proposed by the Kidney Disease Outcomes Quality Initiative (KDOQI).^[8,9]^

GFR was calculated using the Modification of Diet in Renal Disease (MDRD) study 4 variable equation: estimated glomerular filtration rate = 186.3 × (serum standardized creatinine mg/dL)^−1.154^ × age^−0.203^ × (0.742 if female) × (1.21 if black).^[10]^

We also tested association of baseline demographic, clinical, angiographic and procedural characteristics with the incidence of PMI. Hypertension was defined as blood pressure >160/90 mm Hg on repeat measurements, or current use of antihypertensive medications. Hyperlipidemia was defined as documented hyperlipidemia or use of lipid-lowering medications. Smoking status was defined as current smoking or having quit within 6 months before PCI. Coronary lesions were classified according to the American College of Cardiology/American Heart Association (ACC/AHA) into 3 groups (types A, B, C) based on angiographic findings.^[11]^

The primary end point was the incidence of PMI at 8 or 16 hours after elective stent implantation in the control and the CKD groups.

### 2.1 Statistical analysis

Numerical data are presented as mean ± SD in the case of continuous variables with normal distribution, or as median with IQR in the case of not normal distribution. The difference between 2 groups in continuous variables was tested with Student's *t*-test in normally distributed variables or with Mann–Whitney's *U* test in non-normally distributed variables. The difference between 2 groups in categorical variables was tested with Pearson's chi-squared test. A multivariate logistic regression analysis was performed to determine variables independently associated with PMI. All variables that were associated with respective outcome in bivariate analysis (at *P* ≤ 0.1) were included in the multivariate regression. Statistical significance was considered at *P* value <0.05. All statistical analyses were performed by using Statistica for Windows 12.0 software (Statsoft, Tulsa, OK).

## 3 Results

We enrolled 344 patients, among which 242 (70.3%) were males and 102 (29.7%) were females.

**Figure 1:**
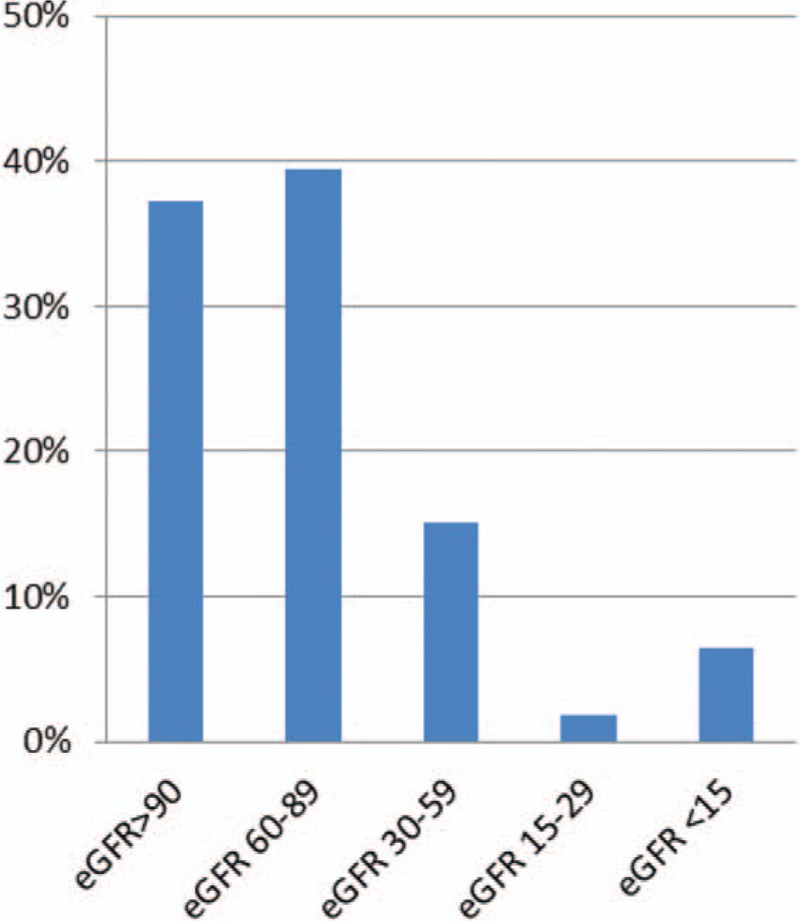
Distribution of patients according to the eGFR (mL/min/1.73 m^2^). eGFR = estimated glomerular filtration rate.

There were 128 (37.2%) patients in the control group with eGFR ≥90 mL/min/1.73 m^2^ and 216 (62.8%) patients in the CKD group with eGFR <90 mL/min/1.73 m^2^. In the CKD group 136 (39.5%) patients had eGFR 60 to 89 mL/min/1.73 m^2^, 52 (15.1%) patients had eGFR 30 to 59 mL/min/1.73 m^2^, 6 (1.8%) patients were with eGFR 15 to 29 mL/min/1.73 m^2^ and 22 (6.4%) patients with eGFR <15 mL/min/1.73 m^2^ (Fig. 1).

Patients in the CKD group were older, more likely to be male and less likely to be current smokers. Other characteristics were similar in the 2 groups. Baseline characteristics for the total study population are given in Table 2.

**Table 2:**
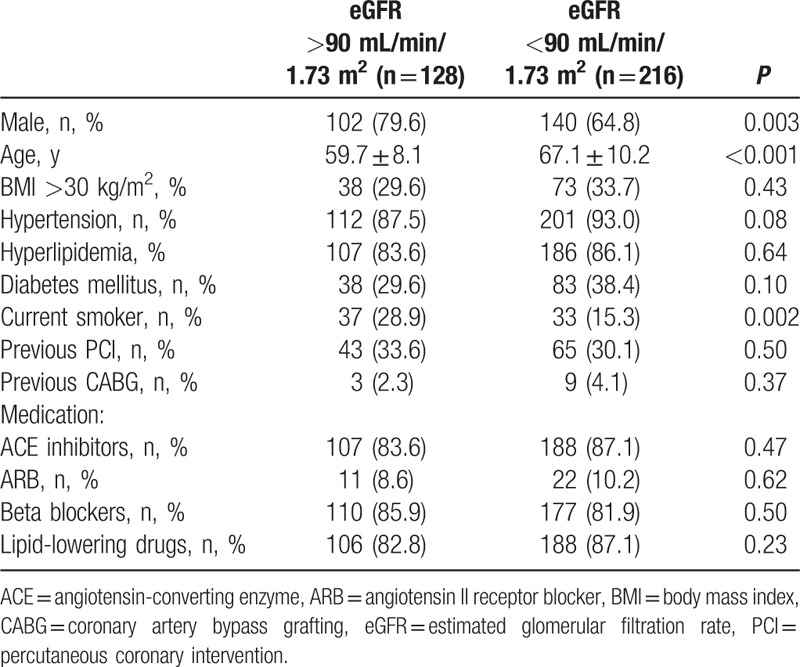
Baseline characteristics of the study participants.

Angiographic and procedural characteristics in both groups were similar. There were no significant differences in lesion locations, type of lesions (AHA/ACC type), and stent procedures between control and study groups. Lesion and procedural characteristics are given in Table 3.

**Table 3:**
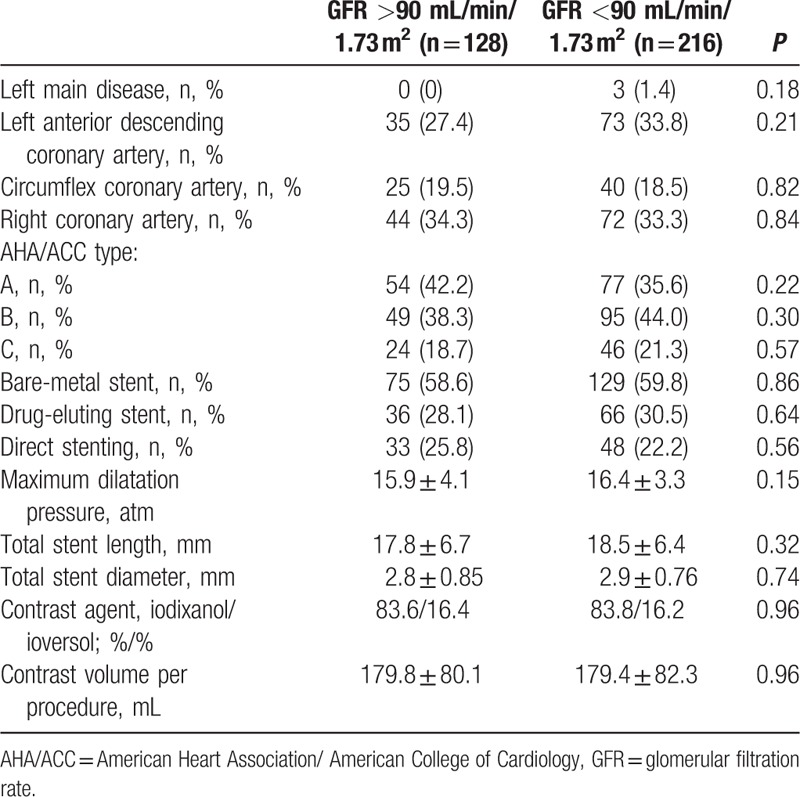
Lesion and procedural characteristics.

**Figure 2:**
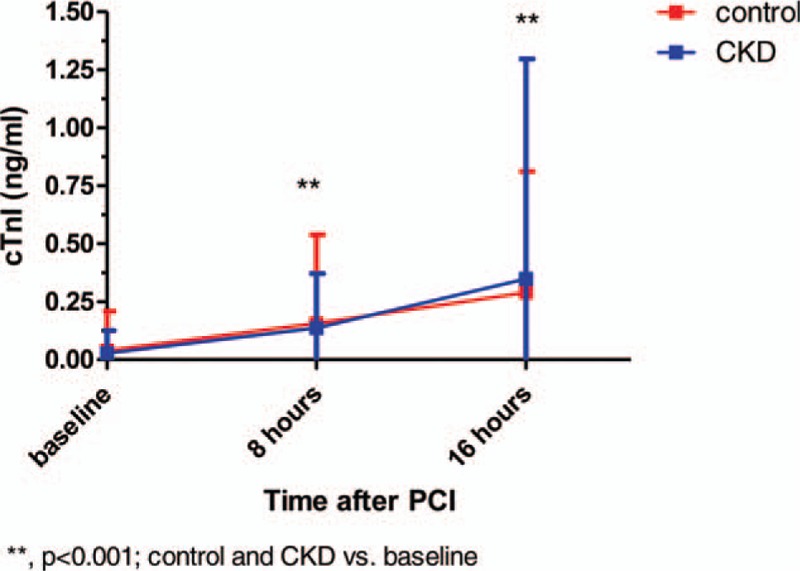
cTnI changes after PCI in the control and CKD groups. CKD = chronic kidney disease, cTnI = cardiac troponin I, PCI = percutaneous coronary intervention.

cTnI in the control and the CKD groups increased 8 and 16 hours after PCI (Fig. 2). However, rise in cTnI was similar in CKD and control groups (Fig. 2).

**Figure 3:**
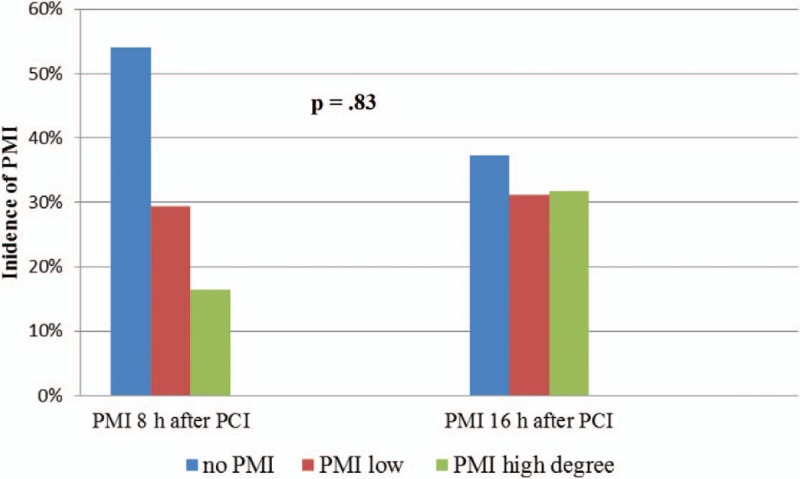
Incidence of PMI among all the patients. eGFR = estimated glomerular filtration rate, PCI = percutaneous coronary intervention, PMI = periprocedural myocardial injury.

Among all patients, the incidence of PMI of high degree 8 hours after elective PCI was 16.5% (57 patients) and 16 hours after elective PCI was 31.7% (109 patients). The incidence of PMI of low degree 8 hours after PCI was 29.4% (101 patients) and 16 hours after PCI 31.1% (107 patients) (Fig. 3).

**Figure 4:**
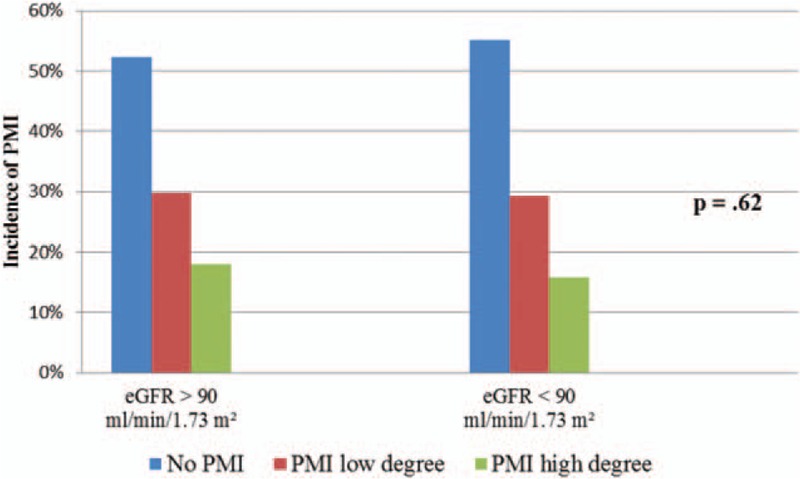
Incidence of PMI 8 h after PCI in patients with CKD and patients without CKD. CKD = chronic kidney disease, eGFR = estimated glomerular filtration rate, PCI = percutaneous coronary intervention, PMI = periprocedural myocardial injury.

**Figure 5:**
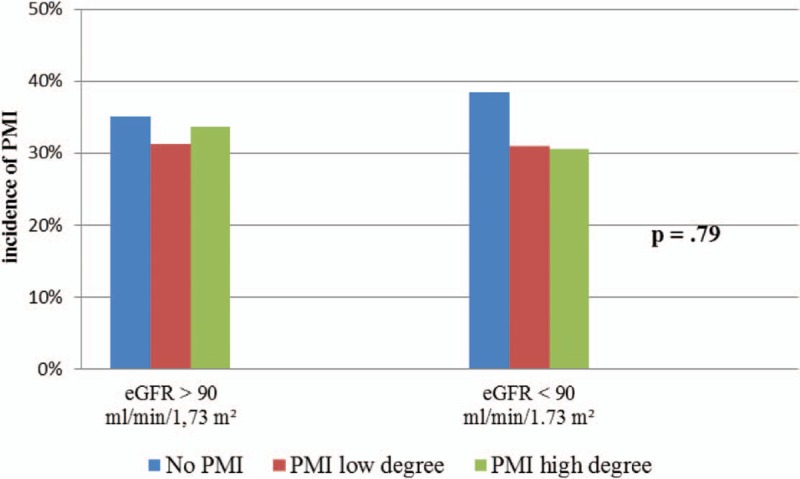
Incidence of PMI 16 h after PCI in patients with CKD and patients without CKD. CKD = chronic kidney disease, eGFR = estimated glomerular filtration rate, PCI = percutaneous coronary intervention, PMI = periprocedural myocardial injury.

There were no significant differences in the incidence of PMI of low or high degree 8 and 16 hours after PCI in patients with CKD and in those without CKD. The incidence of PMI of low degree 8 hours after PCI in the control group was 29.6% (38 patients) and in the CKD group 29.2% (63 patients). On the other hand, 16 hours after PCI a PMI of low degree occurred in 31.2% (40 patients) in the control group and in 31.1% (67 patients) in the CKD group. The incidence of PMI of high degree 8 hours after PCI in the control group was 17.9% (23 patients) and in the CKD group 15.7% (34 patients). High degree PMI 16 hours after PCI occurred in 33.6% (43 patients) in the control group and 30.6% (66 patients) in the CKD group (Figs. 4 and 5).

We further assessed incidence and severity of PMI with respect to CKD burden (i.e., CKD stage). We found no significant differences in the incidence of PMI of high or low degree 8 and 16 hours after PCI in groups according to the eGFR.

**Figure 6:**
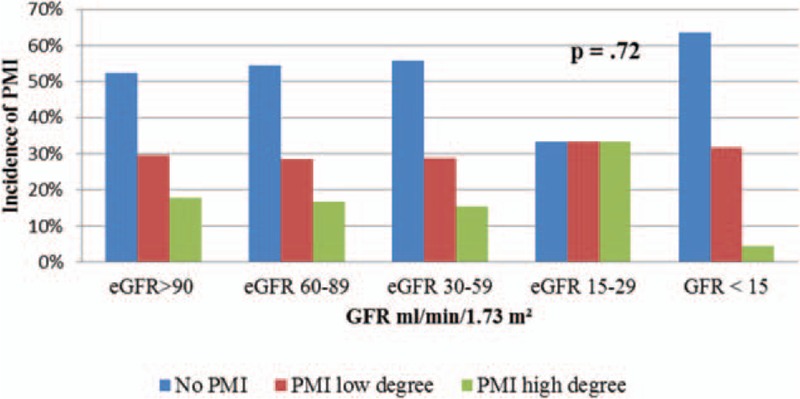
Incidence of PMI 8 h after PCI according to the eGFR. eGFR = estimated glomerular filtration rate, PCI = percutaneous coronary intervention, PMI = periprocedural myocardial injury.

The incidence of PMI of low degree 8 hours after PCI in the CKD group with eGFR 60–89 mL/min/1.73 m^2^ was 28.7% (39 patients) and high degree PMI occurred in 16.9% (23 patients) The incidence of PMI of low degree in the CKD group with eGFR 30–59 mL/min/1.73 m^2^ was 28.8% (15 patients) and the incidence of high degree PMI was 15.4% (8 patients). In the group with eGFR 15–29 mL/min/1.73 m^2^, the incidence of PMI of low degree was 33.3% (2 patients) and PMI of high degree occurred in 33.3% (2 patients). Lastly, in the group with eGFR <15 mL/min/1.73 m^2^, the incidence of PMI of low degree was 31.8% (7 patients) and high degree 4.5% (1 patient) (Fig. 6).

**Figure 7:**
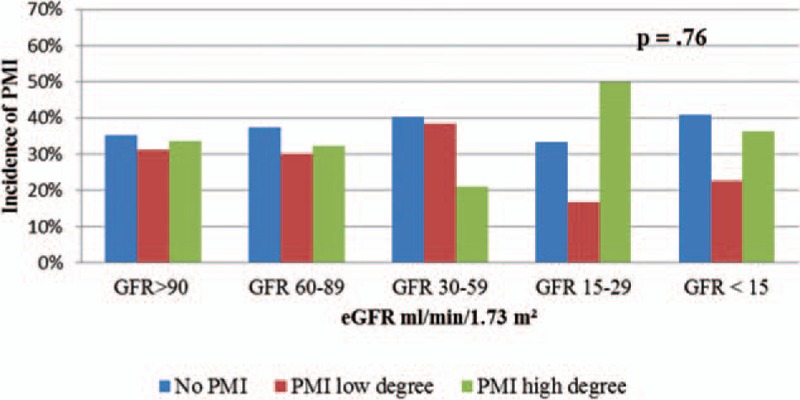
Incidence of PMI 16 h after PCI according to the eGFR. eGFR = estimated glomerular filtration rate, PCI = percutaneous coronary intervention, PMI = periprocedural myocardial injury.

The incidence of a PMI of low degree 16 hours after PCI in CKD group with eGFR 60–89 mL/min/1.73 m^2^ was 30.2% (41 patients) and a PMI of high degree was 32.3% (44 patients). The incidence of PMI of low degree in CKD group with eGFR 30–59 mL/min/1.73 m^2^ was 38.5% (20 patients) and a PMI of high degree occurred in 21.2% (11 patients). In the group with eGFR 15–29 mL/min/1.73 m^2^, the incidence of PMI of low degree was 16.7% (1 patients) and high degree was seen in 50.0% (3 patients). And in the group with GFR <15 mL/min/1.73 m^2^, the incidence of PMI of low degree was 22.7% (5 patients), whereas high degree PMI occurred in 36.4% (8 patients) (Fig. 7).

Patients with eGFR <15 mL/min/1.73 m^2^ had significantly higher levels of C-reactive protein (CRP) before PCI as well as 8 and 16 hours after PCI compared to the control group (Table 4). However, we found no significant differences in CRP levels between control and other CKD groups.

**Table 4:**
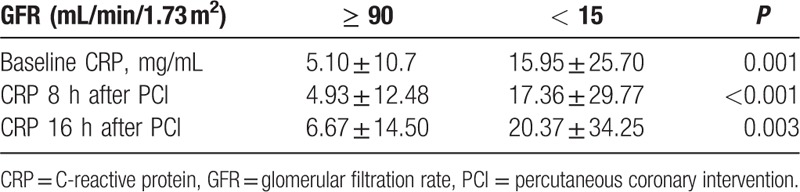
C-reactive protein in patients with glomerular filtration rate <15 mL/min/1.73 m^2^ compared to the control group.

To further evaluate possible influence of other clinical factors on our results, we conducted further statistical analyses. In univariate analyses, predictors of any type of PMI, that is, both of low degree and of high degree, were: smoking history (*P* = 0.02), previous IM (*P* = 0.007), treatment with ACEI (*P* = 0.001), angina pectoris CCS grade IV (*P* = 0.001), multivessel CAD (*P* = 0.01), BMS (*P* = 0.001), stent diameter (*P* = 0.02), and basal cTnI (*P* = 0.008). In univariate analyses, we have also found some variables associated with reduced risk of any type of PMI: treatment with beta blockers (*P* = 0.05), DES (drug-eluting stent, *P* = 0.004), hypertension (*P* = 0.01), and 1 vessel CAD (*P* = 0.007).

In a multiple logistic regression model, angina pectoris CCS grade IV, ACEI therapy and BMS remained significant independent risk factors for PMI after elective PCI (OR 2.159, 95% CI 1.168–3.989, *P* = 0.01, OR 8.725, 95% CI 2.700–28.194, *P* = 0.0002 and OR 2.184, 95% CI 1.199–3.980, *P* = 0.01), whereas hypertension remained an independent protective factor for the development of PMI (OR 0.289, 95% CI 0.116–0.717, *P* = 0.007) (Table 5).

**Table 5:**
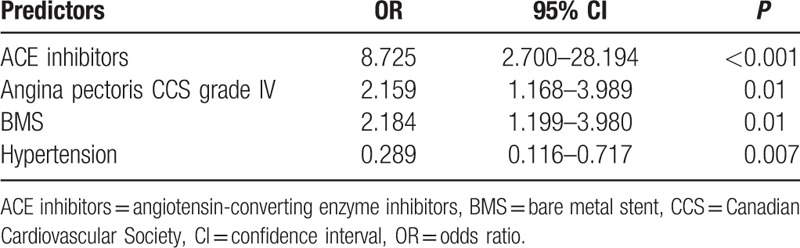
Independent predictors and protective factors for the periproceduralm myocardial injury after elective percutaneous coronary intervention.

## 4 Discussion

Previous studies have shown that a cTn elevation after elective PCI suggests myocardial injury that is related to an increase in the risk of subsequent MI and death.^[12]^ According to the available evidence, baseline cTn elevation in stable coronary disease, is associated with worse long-term prognosis and late cardiovascular mortality.^[13]^ Postprocedural cTn elevation with normal baseline value is associated not only with the late mortality but also with in-hospital mortality.^[14]^ According to those findings, present guidelines recommend routine measurement of cardiac biomarkers both before and after PCI in order to identify high-risk patients.^[7]^ Multiple studies, addressing this issue, have shown the relation between PMI and prognosis, but there were a lot of differences in their design as well as in the definitions of PMI that were used. Such a divergence of definitions, that were used in previous studies, has forced key opinion leaders and cardiology associations to define periprocedural injury. The definition that was brought has been evolving in the past 2 decades. Historically, the definition of periprocedural MI was any rise of cTn over the URL, and there was no difference between PMI and periprocedural MI.^[15]^ The definition from 2007 used cTn elevation ≥ 3×URL as a periprocedural MI and <3×URL was defined as PMI.^[16]^ According to the latest definition endorsed by the ESC, ACCF, AHA, and the WHF, which was used in our study, all patients with postprocedural cTn rise of <5×URL were defined as having a PMI of lower degree. A rise of ≥ 5×URL, in the absence of ischemic, angiographic or imaging findings, was defined as a PMI of a higher degree. Another group of patients that we included, again in accordance with the latest definition, included stable patients with elevated basal cTn values, which were stable or falling. In such patients, PMI of lower degree is defined as any rise of cTn <20% after PCI. Rise of ≥ 20% is defined as a PMI of higher degree. A separate, distinct group of patients is the group with unsuccessful procedure or an angiographic complication that leads to the postprocedural ECG or echocardiographic changes. Those patients are defined by the above definition as having a PCI-related MI (type 4a),^[7]^ and those patients were excluded from our study. We believe that such a periprocedural injury is almost exclusively related to the procedural technique and should be addressed from the technical standpoint rather than trying to explain such an injury by patient's baseline characteristics.

The main finding of our study was that CKD is not a risk factor for occurrence of PMI. We did not find that any level of CKD was associated with higher incidence of PMI (either higher or lower degree), despite the fact that CKD is an important cardiovascular risk factor.^[17]^ This is especially interesting, when knowing that some previous observations have shown that CKD patients had worse outcomes after coronary revascularization.^[18]^ Extensive investigation of the relationship between CKD and PMI after elective PCI was done by Kumagai et al^[18]^ It was found that patients with CKD defined as GFR <60 mL/min/1.73 m^2^ had a higher incidence of PMI compared to patients with GFR >60 mL/min/1.73 m^2^. In that study, GFR was estimated using a formula that was specifically designed for the Japanese population.^[18,19]^ Although an explanation for discrepant results between the present and the Kumagai study is not entirely clear, there are some important methodology differences in relation to PMI definition and study subject inclusion between the 2studies. In the Kumagai report, definition of PMI that was used (>3×URL) was derived from the definition that was contemporary at that moment.^[16]^ Hence, it is not in the accordance with the current recommendations for the PMI diagnosis. Also, it is not clear whether patients with elevated basal cTnT were included and if they were, in what way was the inclusion done. In addition, mathematical manipulation with the baseline levels of cTnT that was done could have influenced the observed incidence of PMI, while by including only the patients with a rise in cTnT to >3× URL, they might have missed those with milder forms of PMI. On the other hand, these patients with mild PMI were included in our study. All those issues may have resulted in the underestimation of PMI occurrence in the Kumagai study, which might have led to the differences in the results of our 2 groups. In addition, we strongly believe that patients with mild PMI should always be included in such studies, as it was shown that even so-called troponin microleaks influence the prognosis after PCI.^[20]^

Limitation of our study was relatively small sample size of stages 4 and 5 CKD patients, so further analysis comprising a larger number of advanced CKD patients is needed to verify our results.

We also found that patients with end-stage CKD had significantly higher levels of inflammation marker CRP before PCI and 8  and 16 hours after PCI compared to patients with preserved GFR. New evidence suggest that inflammation is the most important mechanism of accelerated coronary calcification and endothelial dysfunction in CKD.^[21]^ Thus, CRP should not be observed as a mere inflammation marker, but it can also serve as an indicator of increased cardiovascular morbidity in hemodialysis patients.

By doing further statistical analyses, we tried to find other possible clinical factors that could be associated with the occurrence of PMI. From the previous studies, it is known that aspirin, clopidogrel, beta-blockers, and statins may reduce PMI and patients scheduled for PCI should be pretreated with these drugs.^[22]^ Benefits of statin treatment can be explained not only by their lipid-lowering potential, but also by pleiotropic effects that modify endothelial function, inflammation responses, plaque stability, and thrombus formation.^[23]^ Two randomized trials have analyzed the role of intracoronary beta-blocker propranolol during PCI and showed that propranolol significantly reduced CK-MB, TnT, and clinical end points at 30 days post-PCI.^[24]^ Retrospective studies have shown that intracoronary calcium antagonists nicardipine and verapamil have been successfully used in the treatment of no-reflow following PCI but have failed to reduce the incidence of PMI.^[25]^ Schaefer and group of authors have shown, in a small number of patients, that intracoronary ACEIs during primary PCI suppress reperfusion-associated ventricular arrhythmias and improve coronary blood flow.^[26]^ One recent study showed that chronic therapy with ACEI prior to elective PCI is associated with a 64% relative risk reduction of procedural MI.^[27]^ Unfortunately, the limitations of that study were baseline inequalities between study groups. In our study, quite oppositely, we found that ACEI therapy was associated with an increased incidence of PMI. This is, however, unlikely to be the effect of ACE inhibition itself, but it might reflect some associated clinical characteristics. For example, we found that patients on chronic ACEI had higher baseline prevalence of adverse prognostic features including diabetes, hypertension, previous MI and previous PCI, or CABG (data not shown). In addition, there are several studies that have warned of episodes of profound hypotension during surgical procedures in patients treated chronically with ACEI.^[28]^ Such hypotensive episodes may have lead to coronary hypoperfusion and myocardial injury during PCI. This would further be in agreement with our finding that, in a multivariate logistic regression analysis, hypertension is inversely related to the development of PMI.

Furthermore, we found that BMS implantation significantly increased the incidence of PMI after elective PCI, arguing for use of DES in order to avoid PMI. Looking solely at the CKD patients, there may be several reasons to use DES instead of BMS. Shenoy et al^[29]^ have shown in their study of 436 CKD patients, that use of DES in CKD was safe and effective in the long term, with lower risk of all-cause death, target vessel revascularization (TVR), and the composite of major adverse cardiovascular events (MACE) and similar risk of MI and stent thrombosis (ST) as compared to BMS. Patients with CKD, especially end-stage renal disease, have higher in-stent restenosis rates, and that may be the reason of absolute benefit of DES compared to BMS, knowing that DES significantly suppressed neointimal hyperplasia and reduced the risk of restenosis compared to BMS.^[30,31]^ Unfortunately, there are no large prospective randomized studies comparing DES with BMS regarding the incidence of PMI in both CKD as well as in non-CKD patients. Our finding that BMS is associated with significantly higher incidence of PMI may be explained by higher inflammatory reaction after BMS implantation. BMS implantation is associated with higher risk of in stent restenosis and the crucial mechanism for that is inflammation.^[32]^ On the other side, inflammation plays an important role in the pathogenesis of PMI. Bonz et al^[33]^ have shown that inflammatory markers are elevated in patients with PMI. So, 1 might speculate that BMS implantation in our patients was associated with higher incidence of PMI because of exaggerated inflammatory reaction.

We have also found angina pectoris CCS grade IV as a significant and independent risk factor in the development of PMI. Broda et al^[34]^ have shown, on 300 patients with coronary artery disease, that more extensive coronary lesions correspond to more severe coronary symptoms. They have found that there was significant correlation between coronary symptoms severity (CCS) and anatomic lesions revealed by angiography. Consequently, our finding may reflect more complex underlying coronary anatomy in CCS grade IV patients, which resulted in higher occurrence of PMI.

At the end, we have shown that despite the pharmacologic and technical advances and greater experience with stenting techniques, the incidence of PMI after elective PCI is still quite high. With this regard, our results are similar to the results of the other study groups.^[35,36]^ Large prospective trials showed that post-PCI elevation of cTn (>5× URL), defined in our study as PMI of higher degree, is clinically relevant and has prognostic implications similar to those of spontaneous acute myocardial infarction.^[37,38]^ More recent studies suggested that the degree of cardiovascular risk correlates with the extent of rise of cTn and imaging studies demonstrated that post-procedural levels of cTn represent new irreversible myocardial injury.^[39]^ Additional studies are needed to address strategies to reduce the risk for PMI after PCI.

Another important issue in patients with CKD, that was not subject of the present study, is contrast-induced nephropathy (CIN). Patients with CKD are at increased risk of developing CIN after coronary intervention procedures. Before these procedures, patients should be assessed for risk of CIN using a simple clinical ACEF risk score as a predictor of acute kidney injury after PCI.^[40]^ European Society of Cardiology (ESC) Guidelines recommended that all patients with moderate to severe CKD should receive preventive hydration with isotonic saline, to be started ∼12 hours before angiography and continued for at least 24 hours afterward to reduce the risk of CIN. The guidelines also recommend application of low-osmolar or iso-osmolar contrast media and volume of contrast media should be minimized. The contrast volume should be maintained <4 mL/kg or <350 mL in 1 procedure. Use of high-dose statin before coronary angiography, should be also considered as an additional preventive measure and in patients with severe CKD, ESC Guidelines recommended prophylactic hemofiltration 6 hours before PCI.^[41]^ In our study, we have used in all patients low osmolar contrast media (iodixanol or ioversol), aiming to minimize risk for CIN with all patients being hydrated prior to procedure with isotonic saline, as well.^[42]^

However, we cannot report on the incidence of CIN in the present report, because majority of patients had been discharged from the hospital within 24 hours following the angiography.

## 5 Conclusion

We found no association between PMI occurrence and the presence of CKD. Furthermore, CKD burden (i.e., stratification of patients according to the CKD stage) was also not associated with higher PMI incidence. But, future studies comprising a larger number of advanced CKD patients are needed to verify these findings and further address the issue of both PMI diagnosis and its prognostic implication in patients with CKD.

## References

[1] Collins AJ Foley R Herzog C Excerpts from the United States Renal Data System 2007 annual data report. *Am J Kidney Dis* 2008; 51:S82–S83. 10.1053/j.ajkd.2007.11.001 18086389

[2] Charytan D Mauri L Agarwal A The use of invasive cardiac procedures after acute myocardial infarction in long-term dialysis patients. *Am Heart J* 2006; 152:558–564. 16923431 10.1016/j.ahj.2006.02.021 PMC4398776

[3] Sarnak MJ Levey AS Schoolwerth AC American Heart Association Councils on Kidney in Cardiovascular Disease, High Blood Pressure Research, Clinical Cardiology, and Epidemiology and Prevention Kidney disease as a risk factor for development of cardiovascular disease: a statement from the American Heart Association Councils on Kidney in Cardiovascular Disease, High Blood Pressure Research, Clinical Cardiology, and Epidemiology and Prevention. *Circulation* 2003; 108:2154–2169. 14581387 10.1161/01.CIR.0000095676.90936.80

[4] Ix JH Mercado N Shlipak MG Association of chronic kidney disease with clinical outcomes after coronary revascularization: the Arterial Revascularization Therapies Study (ARTS). *Am Heart J* 2005; 149:512–519. 15864241 10.1016/j.ahj.2004.10.010

[5] Parikh PB Jeremias A Naidu SS Impact of severity of renal dysfunction on determinants of in-hospital mortality among patients undergoing percutaneous coronary intervention. *Catheter Cardiovasc Interv* 2012; 80:352–357. 22566286 10.1002/ccd.23394

[6] Shroff GR Solid CA Herzog CA Long-term survival and repeat coronary revascularization in dialysis patients after surgical and percutaneous coronary revascularization with drug-eluting and bare metal stents in the United States. *Circulation* 2013; 127:1861–1869. 23572500 10.1161/CIRCULATIONAHA.112.001264 PMC3767120

[7] Thygesen K Alpert JS Jaffe AS Third universal definition of myocardial infarction. *Eur Heart J* 2012; 33:2551–2567. 22922414 10.1093/eurheartj/ehs184

[8] Inker LA Astor BC Fox CH KDOQI US commentary on the 2012 KDIGO clinical practice guideline for the evaluation and management of CKD. *Am J Kidney Dis* 2014; 63:713–735. 24647050 10.1053/j.ajkd.2014.01.416

[9] Levey AS de Jong PE Coresh J The definition, classification, and prognosis of chronic kidney disease: a KDIGO Controversies Conference report. *Kidney Int* 2011; 80:17–28. 21150873 10.1038/ki.2010.483

[10] Levey AS Coresh J Greene T Expressing the modification of diet in renal disease study equation for estimating glomerular filtration rate with standardized serum creatinine values. *Clin Chem* 2007; 53:766–772. 17332152 10.1373/clinchem.2006.077180

[11] Ryan TJ Faxon DP Gunnar RM Guidelines for percutaneous transluminal coronary angioplasty. A report of the American College of Cardiology/American Heart Association Task Force on Assessment of Diagnostic and Therapeutic Cardiovascular Procedures (Subcommittee on Percutaneous Transluminal Coronary Angioplasty). *Circulation* 1988; 78:486–502. 2969312 10.1161/01.cir.78.2.486

[12] Prasad A Singh M Lerman A Isolated elevation in troponin T after percutaneous coronary intervention is associated with higher long-term mortality. *J Am Coll Cardiol* 2006; 48:1765–1770. 17084247 10.1016/j.jacc.2006.04.102

[13] Jeremias A Kleiman NS Nassif D Evaluation of Drug Eluting Stents and Ischemic Events (EVENT) Registry Investigators. Prevalence and prognostic significance of preprocedural cardiac troponin elevation among patients with stable coronary artery disease undergoing percutaneous coronary intervention: results from the evaluation of drug eluting stents and ischemic events registry. *Circulation* 2008; 118:632–638. 18645057 10.1161/CIRCULATIONAHA.107.752428

[14] Lindsey JB Marso SP Pencina M EVENT Registry Investigators Prognostic impact of periprocedural bleeding and myocardial infarction after percutaneous coronary intervention in unselected patients: results from the EVENT (evaluation of drug-eluting stents and ischemic events) registry. *JACC Cardiovasc Interv* 2009; 2:1074–1082. 19926047 10.1016/j.jcin.2009.09.002

[15] Alpert JS Thygesen K Antman E Myocardial infarction redefined—a consensus document of The Joint European Society of Cardiology/American College of Cardiology Committee for the redefinition of myocardial infarction. *J Am Coll Cardiol* 2000; 36:959–969. 10987628 10.1016/s0735-1097(00)00804-4

[16] Thygesen K Alpert JS White HD Joint ESC/ACCF/AHA/WHF Task Force for the Redefinition of Myocardial Infarction. Universal definition of myocardial infarction. *J Am Coll Cardiol* 2007; 50:2173–2195. 18036459 10.1016/j.jacc.2007.09.011

[17] Cai Q Mukku VK Ahmad M Coronary artery disease in patients with chronic kidney disease: a clinical update. *Curr Cardiol Rev* 2013; 9:331–339. 24527682 10.2174/1573403X10666140214122234 PMC3941098

[18] Kumagai S Ishii H Amano T Impact of chronic kidney disease on the incidence of peri-procedural myocardial injury in patients undergoing elective stent implantation. *Nephrol Dial Transplant* 2012; 27:1059–1063. 21771758 10.1093/ndt/gfr411

[19] Matsuo S Imai E Horio M Revised equations for estimated GFR from serum creatinine in Japan. *Am J Kidney Dis* 2009; 53:982–992. 19339088 10.1053/j.ajkd.2008.12.034

[20] Milani RV Fitzgerald R Milani JN The impact of micro troponin leak on long-term outcomes following elective percutaneous coronary intervention. *Catheter Cardiovasc Interv* 2009; 74:819–822. 19670308 10.1002/ccd.22160

[21] Arici M Walls J End-stage renal disease, atherosclerosis, and cardiovascular mortality: is C-reactive protein the missing link? *Kidney Int* 2001; 59:407–414. 11168922 10.1046/j.1523-1755.2001.059002407.x

[22] Babu GG Walker JM Yellon DM Peri-procedural myocardial injury during percutaneous coronary intervention: an important target for cardioprotection. *Eur Heart J* 2011; 32:23–31. 21037252 10.1093/eurheartj/ehq393

[23] Briguori C Colombo A Airoldi F Statin administration before percutaneous coronary intervention: impact on periprocedural myocardial infarction. *Eur Heart J* 2004; 25:1822–1828. 15474697 10.1016/j.ehj.2004.07.017

[24] Wang FW Osman A Otero J Distal myocardial protection during percutaneous coronary interventions with an intracoronary beta-blocker. *Circulation* 2003; 107:2914–2919. 12771007 10.1161/01.CIR.0000072787.25131.03

[25] Arora S Alfayoumi F Khawaja AT Effects of intracoronary nicardipine on cardiac enzymes after elective percutaneous coronary intervention. *Clin Cardiol* 2009; 32:315–320. 19569065 10.1002/clc.20580 PMC6653674

[26] Schaefer U Kurz T Bonnemeier H Intracoronary enalaprilat during angioplasty for acute myocardial infarction: alleviation of postischaemic neurohumoral and inflammatory stress? *J Intern Med* 2007; 261:188–200. 17241184 10.1111/j.1365-2796.2006.01757.x

[27] Aleti S Bansal D Agrawal M Effect of chronic angiotensin converting enzyme inhibitor therapy on myocardial injury in patients undergoing percutaneous coronary interventions. *J Invasive Cardiol* 2011; 23:72–75. 21297204

[28] Nabbi R Woehlck HJ Riess ML Refractory hypotension during general anesthesia despite preoperative discontinuation of an angiotensin receptor blocker. *F1000Research* 2013; 2:12. 24358842 10.12688/f1000research.2-12.v1 PMC3752674

[29] Shenoy C Boura J Orshaw P Drug-eluting stents in patients with chronic kidney disease: a prospective registry study. *PLoS One* 2010; 5:e15070. 21124771 10.1371/journal.pone.0015070 PMC2993936

[30] Halkin A Selzer F Marroquin O Clinical outcomes following percutaneous coronary intervention with drug-eluting vs. bare-metal stents in dialysis patients. *J Invasive Cardiol* 2006; 18:577–583. 17197706

[31] Resmini C Di Cuia M Ballocca F Short and long term outcome of percutaneous coronary intervention with drug eluting stent and bare metal stent in patients with chronic kidney disease. *Minerva Cardioangiol* 2012; 60:573–580. 23147435

[32] Jeong YH Hong MK Lee CW Impact of significant chronic kidney disease on long-term clinical outcomes after drug eluting stent versus bare metal stent implantation. *Int J Cardiol* 2008; 125:36–40. 17521753 10.1016/j.ijcard.2007.02.026

[33] Bonz AW Lengenfelder B Jacobs M Cytokine response after percutaneous coronary intervention in stable angina: effect of selective glycoprotein IIb/IIIa receptor antagonism. *Am Heart J* 2003; 145:693–699. 12679767 10.1067/mhj.2003.65

[34] Broda K Pawlus E Bulara A Severity of angina pectoris and coronary angiographic imaging. *Przegl Lek* 1996; 53:713–716. 9091947

[35] Novack V Pencina M Cohen DJ Troponin criteria for myocardial infarction after percutaneous coronary intervention. *Arch Intern Med* 2012; 172:502–508. 22371874 10.1001/archinternmed.2011.2275

[36] Testa L Van Gaal WJ Biondi Zoccai GG Myocardial infarction after percutaneous coronary intervention: a meta-analysis of troponin elevation applying the new universal definition. *QJM* 2009; 102:369–378. 19286891 10.1093/qjmed/hcp005

[37] Nienhuis MB Ottervanger JP Dikkeschei B Prognostic importance of troponin T and creatine kinase after elective angioplasty. *Int J Cardiol* 2007; 120:242–247. 17182137 10.1016/j.ijcard.2006.10.002

[38] Stone GW Mehran R Dangas G Differential impact on survival of electrocardiographic Q-wave versus enzymatic myocardial infarction after percutaneous intervention: a device-specific analysis of 7147 patients. *Circulation* 2001; 104:642–647. 11489768 10.1161/hc3101.093902

[39] Cuculi F Lim CC Banning AP Periprocedural myocardial injury during elective percutaneous coronary intervention: is it important and how can it be prevented? *Heart* 2010; 96:736–740. 20448123 10.1136/hrt.2009.186189

[40] Andò G Morabito G de Gregorio C The ACEF score as predictor of acute kidney injury in patients undergoing primary percutaneous coronary intervention. *Int J Cardiol* 2013; 168:4386–4387. 23711447 10.1016/j.ijcard.2013.05.049

[41] Windecker S Kolh P Alfonso F 2014 ESC/EACTS Guidelines on myocardial revascularization: The Task Force on Myocardial Revascularization of the European Society of Cardiology (ESC) and the European Association for Cardio-Thoracic Surgery (EACTS) Developed with the special contribution of the European Association of Percutaneous Cardiovascular Interventions (EAPCI). *Eur Heart J* 2014; 35:2541–2619. 25173339 10.1093/eurheartj/ehu278

[42] Weisbord SD Palevsky PM Prevention of contrast-associated acute kidney injury: what should we do? *Am J Kid Dis* 2016; 68:518–521. 27233380 10.1053/j.ajkd.2016.05.005

